# Human umbilical cord-derived mesenchymal stromal cells ameliorate aging-associated skeletal muscle atrophy and dysfunction by modulating apoptosis and mitochondrial damage in SAMP10 mice

**DOI:** 10.1186/s13287-022-02895-z

**Published:** 2022-06-03

**Authors:** Limei Piao, Zhe Huang, Aiko Inoue, Masafumi Kuzuya, Xian Wu Cheng

**Affiliations:** 1 Department of Human Life Cord Applied Cell Therapy, Graduate School of Medicine, Nagoya University, Nagoya, Aichi-ken 466-8550 Japan; 2 Department of Cardiology and Hypertension, Yanbian University Hospital, Yanji, 133000 Jilin People’s Republic of China; 3 Department of Community Healthcare and Geriatrics, Nagoya University Graduate School of Medicine, Nagoya, Aichi-ken 466-8550 Japan; 4 Institute of Innovation for Future Society, Nagoya University Graduate School of Medicine, Nagoya, Aichi-ken 466-8550 Japan

**Keywords:** Umbilical cord-derived mesenchymal stromal cell, Sarcopenia, Apoptosis, Inflammation, Mitochondria, SAMP10

## Abstract

**Background:**

Skeletal muscle mass and function losses in aging individuals are associated with quality of life deterioration and disability. Mesenchymal stromal cells exert immunomodulatory and anti-inflammatory effects and could yield beneficial effects in aging-related degenerative disease.

**Methods and results:**

We investigated the efficacy of umbilical cord-derived mesenchymal stromal cells (UC-MSCs) on sarcopenia-related skeletal muscle atrophy and dysfunction in senescence-accelerated mouse prone 10 (SAMP10) mice. We randomly assigned 24-week-old male SAMP10 mice to a UC-MSC treatment group and control group. At 12 weeks post-injection, the UC-MSC treatment had ameliorated sarcopenia-related muscle changes in performance, morphological structures, and mitochondria biogenesis, and it enhanced the amounts of proteins or mRNAs for myosin heavy chain, phospho-AMP-activated protein kinase, phospho-mammalian target of rapamycin, phospho-extracellular signal-regulated kinase1/2, peroxisome proliferator-activated receptor-γ coactivator, GLUT-4, COX-IV, and hepatocyte growth factor in both gastrocnemius and soleus muscles, and it reduced the levels of proteins or mRNAs for cathepsin K, cleaved caspase-3/-8, tumor necrosis factor-α, monocyte chemoattractant protein-1, and gp91^phox^ mRNAs. The UC-MSC treatment retarded mitochondria damage, cell apoptosis, and macrophage infiltrations, and it enhanced desmin/laminin expression and proliferating and CD34^+^/Integrin α_7_^+^ cells in both types of skeletal muscle of the SAMP10 mice. In vitro, we observed increased levels of HGF, PAX-7, and MoyD mRNAs at the 4th passage of UC-MSCs.

**Conclusions:**

Our results suggest that UC-MSCs can improve sarcopenia-related skeletal muscle atrophy and dysfunction via anti-apoptosis, anti-inflammatory, and mitochondrial biogenesis mechanisms that might be mediated by an AMPK-PGC1-α axis, indicating that UC-MSCs may provide a promising treatment for sarcopenia/muscle diseases.

## Background

Sarcopenia, which was recently recognized as a chronic disease by the World Health Organization, is a degenerative loss of skeletal muscle mass and function with aging that results in disability and a reduction in an individual's quality of life [1, 2]. The causes of sarcopenia are multifactorial and include environmental and biological factors, especially inflammatory cytokines, apoptosis, and mitochondrial dysfunction in the skeletal muscle [3–5]. In this context, many therapeutic strategies have been tested in attempts to counteract the aging-related losses of mobility and locomotion.

Although physical exercise has been considered the most effective approach to prevent aging-associated muscle atrophy [5–7], exercise is often inefficient or impractical for older individuals with decreased functional capacities [8]. Several pharmacological therapies (including growth and sex hormones) have been designed to counteract the development of sarcopenia, focusing on interventions that administer growth or sex hormones; however, the therapies have frequently involved side effects [9–11]. The development of new alternatives based on the pathophysiological mechanisms of aging-related muscle atrophy is thus desired.

Mesenchymal stromal cells (MSCs) are a heterogeneous subset of stromal cells that can be isolated from various adult tissues (e.g., bone marrow, adipose tissue, and the umbilical cord and its blood) for tissue regeneration and aging-related disease treatments [12–14]. MSCs have been shown to limit hyperinflammatory processes and promote tissue repair, thus preventing age-related diseases such as muscle atrophy, possibly by the modulation of paracrine growth and/or anti-inflammatory signaling (for a review, see ref. 15). As the umbilical cord is considered medical waste and the access to umbilical cord-derived MSCs (UC-MSCs) has not been encumbered with ethical problems, UC-MSCs are becoming a promising tool in regenerative medicine for their distinct capacity of self-renewal while maintaining their multilineage differentiation into adipocytes, osteocytes, chondrocytes, neurons, and hepatocytes [16–18].

In clinical applications, UC-MSCs have been a versatile candidate because of their noninvasive, painless procurement and collection procedures [19, 20]. These cells also possess a unique combination of prenatal and postnatal stem cell properties and have higher proliferative potential and lower immunogenicity with allogeneic sources than other the commonly used MSCs such as bone marrow-derived MSCs [16]. A dose-dependent protective effect of human UC-MSCs on brain dysfunction via microglial immunomodulation was observed in a mouse model of neonatal stroke [12]. Zhu and colleagues also demonstrated that UC-MSCs protected against a lipopolysaccharide-induced acute lung injury via immune regulation and paracrine factors [21]. However, the therapeutic benefits and potential molecular mechanism of UC-MSCs on aging-related muscle atrophy have not been investigated. Our present study revealed therapeutic effects of human UC-MSCs against the loss of muscle mass and function in senescence-accelerated mouse prone 10 (SAMP10) mice.

## Materials and methods

### Animal care and use

Eight-week-old male SAMP10 mice (SAMP10/TaSlc) were obtained from Japan SLC (Hamamatsu, Japan). They were fed a standard diet and housed one per cage under standard conditions (23 ± 1 °C, 50 ± 5% humidity) for 16 weeks, with a 12-h light/dark cycle in a viral pathogen-free facility at the Laboratory Animal Center of the Nagoya University Graduate School of Medicine. All experiments were approved by the Ethics Committee of Nagoya University. The animal study protocol (No. 31442) was carried out in accord with the approved guidelines.

### Preparation of UC-MSCs

UC-MSCs were provided by the Cord Blood/Umbilical Cord Bank of the Affiliated Hospital of Institute of Medical Sciences, University of Tokyo (IMSUT CORD, Tokyo). Cells were rapidly thawed in a 37 °C water bath just before use, without washing. Subsequent special serum-free CiMS-BM medium (A2G00P05C, Nipro, Osaka, Japan) for human UC-MSCs was used as a conditional medium. The medium was changed every 3 days, and cells were sub-cultured when they reached 90% confluence with the seeding density of 10^4^ cells/cm^2^. After they became confluent, adherent cells were trypsinized and replated (passage 1) and allowed to progress to passage 4.

### UC-MSC treatments and tissue collections

For the in vivo experiments, fourth-passage cells were concentrated to a 150-μl volume with serum-free culture medium by a relatively slow centrifugal speed. We randomly assigned 24-week-old male SAMP10 mice to two groups: control (Cont, n = 7) and UC-MSC treatment (Cell, n = 8). We slowly infused (with a 30-ga. needle via the tail vein with the mouse under body restraint) 150 μl of culture medium alone (Cont) or with 1 × 10^6^ cells/150 μl (Cell). The mice were then subjected to muscle function (grip strength and endurance capacity) assessments at the indicated timepoints.

At 36 weeks after the cell injection, after a muscle performance test, the mice were anesthetized with an intraperitoneal injection of pentobarbital sodium (50 mg/kg), and blood and tissue samples were isolated. The soleus and gastrocnemius muscles were isolated and kept in liquid nitrogen (for the protein assay) or in RNAlater solution (for the gene assay). After being immersed in fixative at 4 °C, the muscle samples were embedded in OCT compound and stored at − 20 °C for the morphological investigations.

Evaluation of grip strength.

The grip strength of the mice was studied as described [5]; in brief, the mouse's forelimbs were placed on the limb grip of a small-animal grip-strength meter (Columbus, Largo, FL, USA), and then the mouse's tail was gently pulled in the opposite direction. We calculated the maximum value of the grip force before it released its grip. The grip strength was recorded > 5 times and averaged as the expression of grip strength for each mouse at 4, 8, and 12 weeks after the cell injection.

### Evaluation of muscle endurance capacity

A motorized rodent treadmill (TMS-M4, MELQUEST: Tokyo Engineering) was used to monitor the running endurance capacity of the mice. For the preliminary training program, mice at day 0 before the cell injection were put on the treadmill at an inclination of 0°: warm-up (5 min), 7 m/min; exercise (35 min), 17 m/min; cool-down (5 min), 7 m/min. For the evaluation of endurance, the mice were put on the treadmill at 4, 8, and 12 weeks after the cell injection, and the warm-up was started at 6 m/min with the treadmill's tilt angle at 0°. After 5 min, the tilt angle of the treadmill was set to 10° and the speed was gradually increased by 2 m/min every 2 min until it reached the maximum speed of 20 m/min and then maintained at the maximum speed as described [5].

The evaluations of the workload and running distance were stopped when the mouse gave up moving for > 10 s. Each mouse's running distance is expressed as a result of the running the time.

### Assays of morphometry and immunohistochemistry

Serial cross-cryosections (4 μm thick) of the soleus and gastrocnemius muscles were collected at rate of 3–5 sections every 45 μm and stained with hematoxylin and eosin (H&E). The area of the muscle fibers was calculated in four chosen microscopic fields from 3 to 5 different sections in each muscle tissue block and averaged for each animal. For the evaluation of fibrosis, Masson's trichrome staining was performed as described [22]. For the immunostaining, corresponding slides were incubated with a mouse monoclonal antibody (mAb) against macrophages (i.e., CD68, clone-KP1; cat. no. ab955, Abcam, Cambridge, UK), a mouse mAb against proliferating cell nuclear antigen (PCNA; cat. no. NA03, Merck Millipore, Darmstadt, Germany), or a rabbit polyclonal antibody (pAb) against human dystrophin (cat. no. 12715–1-AP; Proteintech, Chicago, IL). The sections were then visualized with an ABC substrate kit (SK-4400, Vector Laboratories, Burlingame, CA) in accord with the manufacturer's instructions.

Double immunofluorescence was carried out using a mouse mAb against desmin (1:100; Clone 33, Dako, Carpinteria, CA) and a rabbit pAb against laminin 5 (1:100; BS-6713R, Bioss Antibodies, Woburn, MA) or a goat pAb to integrin-α7 (1:100; sc-27706, Santa Cruz Biotechnology, Santa Cruz, CA), and a rabbit mAb to CD34 (1:100; ab110643, Abcam). The muscle sections were visualized using Zenon rabbit and mouse IgG labeling kits (1:200; Molecular Probes, Eugene, OR) as described [22]. The muscle slides were mounted in glycerol-based Vectashield medium (Vector Laboratories). For negative control staining, the primary antibodies were replaced with Zenon-labeled rabbit or mouse IgG.

### Immunoblotting analysis

Total protein was isolated with the use of a RIPA lysis buffer, and the proteins were then Western-blotted against antibodies for total mammalian target of rapamycin (mTOR, #4517), phospho-mTOR^er2448^ (#2971), total extracellular signal-regulated kinase1/2 (Erk1/2, #9107), phospho-Erk1/2^thr202/tyr204^ (p-Erk1/2^thr202/tyr204^, #4377), total adenosine monophosphate (AMP)-activated protein kinase alpha (AMPKα, #2793), phospho-AMPKα^thr172^ (p-AMPKα^thr172^, #2531), cleaved caspase-8 (C-casp-3, #9429), cleaved caspase-3 (C-casp-8, #9661; Cell Signaling Technology), peroxisome proliferator-activated receptor-γ coactivator1-α (PGC1-α, ab54481), rabbit pAb against slow myosin heavy chain (sMHC, ab11083; Abcam) and glyceraldehyde 3-phosphate dehydrogenase (GAPDH, sc-20357, Santa Cruz Bio-technology) (1:1000 for each antibody).

### Gene expression assay

Total RNA was isolated from the muscles and lysates with a RNeasy Fibrous Tissue Mini-Kit (Qiagen, Hilden, Germany), and mRNA was reverse-transcribed to cDNA with an RNA PCR Core kit (Applied Biosystems, Foster City, CA). A quantitative real-time polymerase chain reaction (RT-PCR) assay was applied to evaluate using the ABI 7300 RT-PCR system with Universal PCR Master Mix (Applied Biosystems). All assays were performed in triplicate. The sequences of the primers for cytochrome c oxidase subunit 4 (COX-IV), glucose transporter-4 (GLUT-4), PGC1-α, hepatocyte growth factor (HGF), paired box-7 (PAX-7), myogenic differentiation antigen (MyoD), tumor necrosis factor (TNF)-α, monocyte chemoattractant protein-1 (MCP-1), gp91^phox^, vascular endothelial growth factor (VEGF), toll-like receptor-2 (TLR-2), cathepsin K (CatK), and GAPDH genes are shown in Table 1. Targeted gene transcriptions were normalized against that of the corresponding GAPDH.

**Table 1:** Primer sequences used for the quantitative real-time PCR

hPAX7-F hPAX7-R	GAAAACCCAGGCATGTTCAG GCGGCTAATCGAACTCACTAA
hMyoD-F hMyoD-R	CACTACAGCGGCGACTCC TAGGCGCCTTCGTAGCAG
hHGF-F hHGF-R	TCTGCATTGCACTTATGCTGA AAAGGGCGATCTAGTGATGGA
mHGF-F mHGF-R	GGCAAGGTGACTTTGAATGA CACATGGTCCTGATCCAATC
mPGC1-α-F mPGC1-α-R	CCGAGAATTCATGGAGCAAT TTTCTGTGGGTTTGGTGTGA
mCOX-IV-F mCOX-IV-R	AGCTGAGCCAAGCAGAGAAG AATCACCAGAGCCGTGAATC
mGLUT-4-F mGLUT-4-R	GACGGACACTCCATCTGTTG GCCACGATGGAGACATAGC
mTNF-α-F mTNF-α-R	AGGCTGCCCCGACTACGT AGGCTGCCCCGACTACGT
mMCP-1-F mMCP-1-R	GCCCCACTCACCTGCTGCTACT CCTGCTGCTGGTGATCCTCTTGT
mgp91^phox^-F mgp91^phox^-R	ACTTTCCATAAGATGGTAGCTTGG GCATTCACACACCACTCAACG
mVEGF-F mVEGF-R	TGTACCTCCACCATGCCAAGT TGGAAGATGTCCACCAGGGT
mTLR-2-F mTLR-2-R	AAGAAGCTGGCATTCCGAGGC CGTCTGACTCCGAGGGGTTGA
mCatK-F mCatK-r	AGCAGGCTGGAGGACTAAGGT TTTGTGCATCTCAGTGGAAGACT
mGAPDH-F mGAPDH-R	ATGTGTCCGTCGTGGATCTGA ATGCCTGCTTCACCACCTTCT
hGAPDH-F hGAPDH-R	GGACTTCGAGCAGGAGATGG GCACCGTGTTGGCGTAGAGG

COX-IV, cytochrome c oxidase subunit 4; GAPDH, glyceraldehyde 3-phasphate dehydrogenase; GLUT-4, glucose transporter-4; HGF, hepatocyte growth factor; MCP-1, monocyte chemoattractant protein-1; MyoD, myogenic differentiation antigen; PAX-7, paired box-7; PGC1-α, peroxisome proliferator-activated receptor-γ coactivator1-α; TNF-α, tumor necrosis factor-α; TLR-2, toll-like receptor-2; CatK, cathepsin K; VEGF, vascular endothelial growth factor

### Electron microscopy

Skeletal muscle tissues were cut into approx. 1-mm^3^ pieces and fixed for 24 h with 0.16 M phosphate-buffered saline containing 2% glutaraldehyde (pH 7.2) and then for 1 h with 1% osmium tetroxide as described.[5] The fixed muscles were dehydrated by a graded series of ethanol solutions before being exposed to propylene oxide and embedded in Epon. The sections were cut at a thickness of 60–70 nm, stained with lead citrate and uranyl acetate, and studied using a transmission electron microscope (JEM-1400, JEOL, Tokyo) operating at 100 kV.

The quantitation of mitochondrial size and number was done at a magnification of 15.000 × by counting the corresponding number of pixels using Adobe Photoshop CS5 software. A total of 50–70 mitochondrial cross sections from 5 to 7 sections were calculated and averaged for each mouse, and distribution diagrams were obtained separately for each group (Cell, Cont).

### Terminal deoxynucleotidyl transferase-mediated dUTP nick end labeling (TUNEL) staining

The apoptotic cells in the muscles were evaluated by a TUNEL staining kit (cat. 11,684,795,910, Roche, Indianapolis, IN). The prepared cross-cryosections of muscle were blocked with 0.1% bovine albumin serum and then treated with TUNEL staining reagents according to the manufacturer's instructions.

UC-MSC culture assay.

For the in vitro experiments, primary UC-MSCs from three donors (n = 3) were obtained and cultured as described above. The differentiation potential and cell surface molecules of the expanded UC-MSCs were validated. The morphology of the UC-MSCs was observed under a light microscope (EVOS FL Auto2, Invitrogen, Carlsbad, CA).

### Statistical analyses

The values are expressed as means ± standard error of the mean (SEM). Student's t tests were applied for comparisons of two groups. Multiple comparisons (three or more groups) of parametric data were performed by a one-way analysis of variance (ANOVA) followed by Tukey post hoc tests. The two parameters endurance and grip strength were subjected to a two-way repeated-measures ANOVA and Bonferroni post hoc tests. SPSS software ver. 19.0 (SPSS, Chicago, IL) was used. P values < 0.05 were considered significant.

## Results

### UC-MSCs ameliorated the muscle loss and dysfunction in SAMP10 mice

**Fig. 1:**
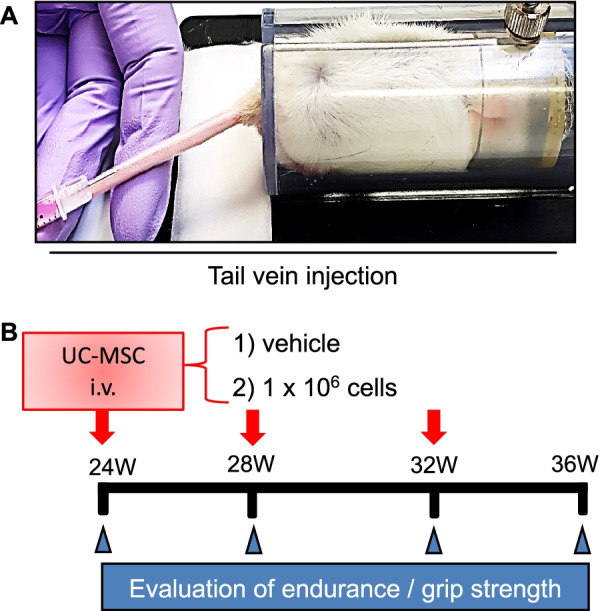
The protocol of the umbilical cord-derived mesenchymal stem cells (UC-MSCs) treatment by tail vein injection and the in vivo cell tracking. **A** The mouse tail vein injection model. **B** Schematic representation of the cell treatment program

**Fig. 2:**
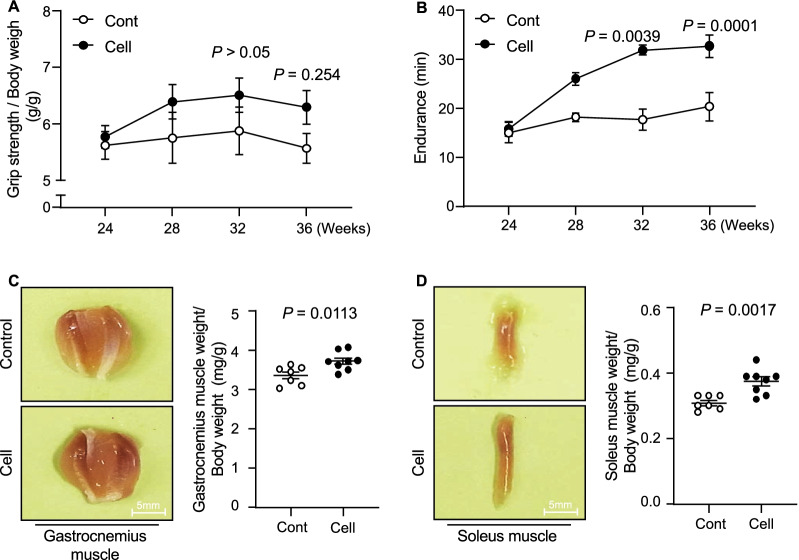
The effects of cell treatment on muscle function at 3 months post-cell treatment. **A**, **B** Grip strength/body weight (BW) and endurance were recorded in the control (Cont, n = 7) and UC-MSC treatment (Cell, n = 8) groups at the indicated timepoints. **C**, **D** The ratios of soleus muscle to BW and the ratios of gastrocnemius to BW were calculated in the Cont (n = 7) and Cell (n = 8) groups at 36 weeks of age in both groups. Data are mean ± SEM

**Fig. 3:**
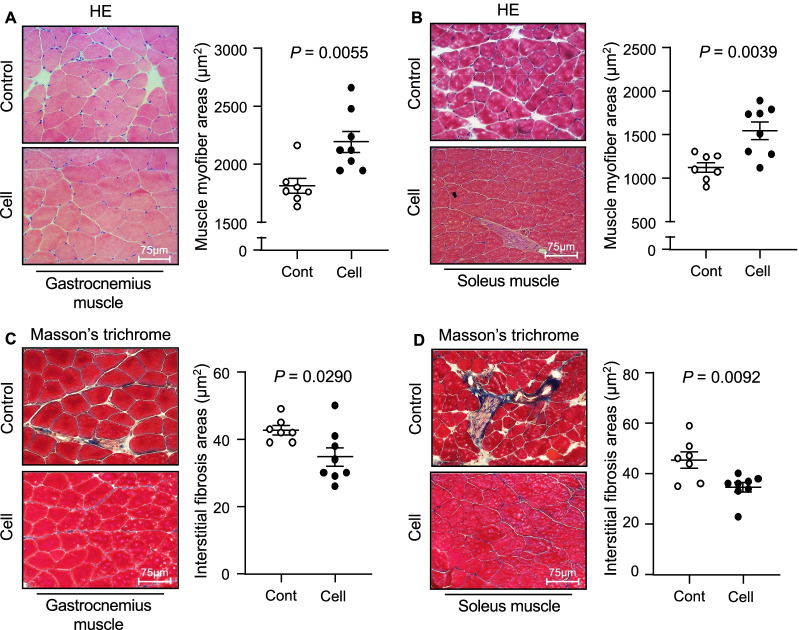
The effects of cell treatment on muscle mass, muscle function, and myofiber size. **A**, **B** Representative hematoxylin and eosin (HE) staining and quantitative data showing the myofiber size in both muscles. Scale bar: 75 μm. **C**, **D** Representative images of Masson's trichrome staining of gastrocnemius muscle (**C**) and soleus muscle (**D**). Scale bar: 75 μm. Data are mean ± SEM

To examine the impact of UC-MSCs on aging-associated muscle morphological changes and dysfunction, we administered an injection of 1 × 10^6^ UC-MSCs to SAMP10 mice and measured their grip strength and endurance at the indicated timepoints (Fig. 1). Although there were no significant differences between the control and UC-MSC-treated groups at 2 months after the injection intervention, the phenotype of skeletal muscle function (both grip strength and endurance) was clearly enhanced in the UC-MSC-treated mice at 32 and/or 36 weeks post-intervention (Fig. 2A,B). In agreement with these phenotype findings, the UC-MSC-treated group had significantly higher ratios of gastrocnemius muscle to body weight (BW) and soleus muscle to BW compared to the control group (Fig. 2C,D). Likewise, the UC-MSCs significantly increased the myofiber size (Fig. 3A,B) and decreased the degree of interstitial myofibrosis (Fig. 3C,D).

### AMPK signaling activation is involved in UC-MSC-mediated muscle benefits

**Fig. 4:**
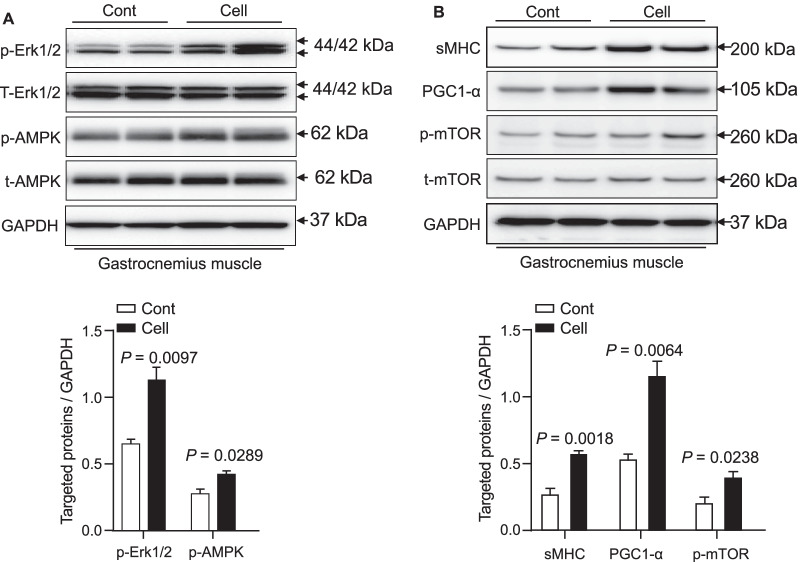
The effects of cell treatment on targeted protein expression and phosphorylation in 36-week-old mice. **A**, **B** Representative immunoblots show the levels of the targeted protein expression or phosphorylation in the gastrocnemius muscle. Quantitative analysis of Western blots for the levels of p-Erk1/2 and p-AMPKα (**A**) and sMHC, PGC1-α, and p-mTOR (**B**) and in the two groups. The expression level of each targeted protein was normalized with a Western blot antibody to GAPDH. Data are mean ± SEM (n = 3)

**Fig. 5:**
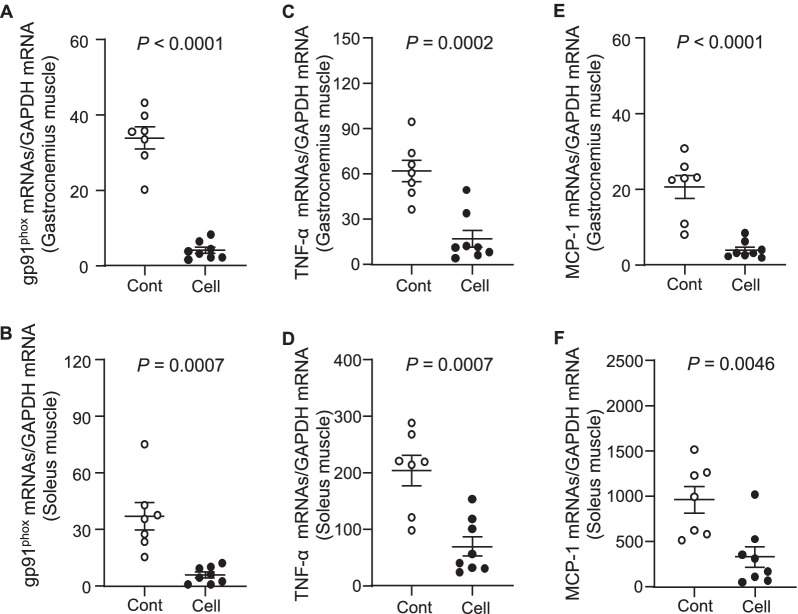
Effects of cell treatment on muscle oxidative stress and inflammation in 36-week-old mice. **A**–**F**: The mRNA expression levels of gp91phox (A,B), TNF-α (C,D), MCP-1 (E,F) in gastrocnemius muscle and soleus muscle were measured by quantitative RT-PCR. Data are mean ± SEM (Cont, n = 7; Cell, n = 8)

A close association between the inactivation of AMPK and various types of muscle dysfunction has been indicated in experimental and human chronic disease [23]. To further examine the consequences of UC-MSC-induced molecular changes in our present experimental conditions, we analyzed the extracted muscles to investigate the role of the AMPK signaling pathway. As shown in Fig. 4, we observed that the UC-MSC-treated mice had significantly increased levels of p-AMPK protein in their gastrocnemius muscle. Conversely, the quantitative RT-PCR data demonstrated that the administration of UC-MSC suppressed the gp91^phox^ gene expression in both the gastrocnemius and soleus muscles (Fig. 5A,B).

The phosphoinositide 3-kinase (PI3K)/mTOR is one of the intracellular pathways used to direct metabolism and growth [24]. In the present study, the representative images and the quantification by Western blotting (WB) indicated that the UC-MSC treatment not only enhanced the phosphorylation of p-mTOR protein; it also resulted in substantial increases of p-Erk1/2 and fibers expressing slow MHC proteins in the gastrocnemius muscles compared to the control SAMP10 mice (Fig. 4A,B).

### UC-MSCs improved muscle mitochondrial biogenesis and dysfunction

**Fig. 6:**
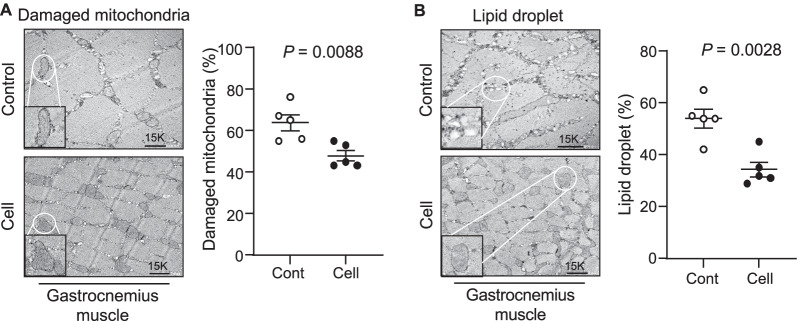
Cell treatment ameliorated the aging-related mitochondria and lipid droplets. **A**, **B**: Representative electron microscopy images showing a relatively preserved mitochondrial configuration (**A**) as well as a small amount of lipid droplets (**B**). Data are mean ± SEM (Cont, n = 7; Cell, n = 8)

**Fig. 7:**
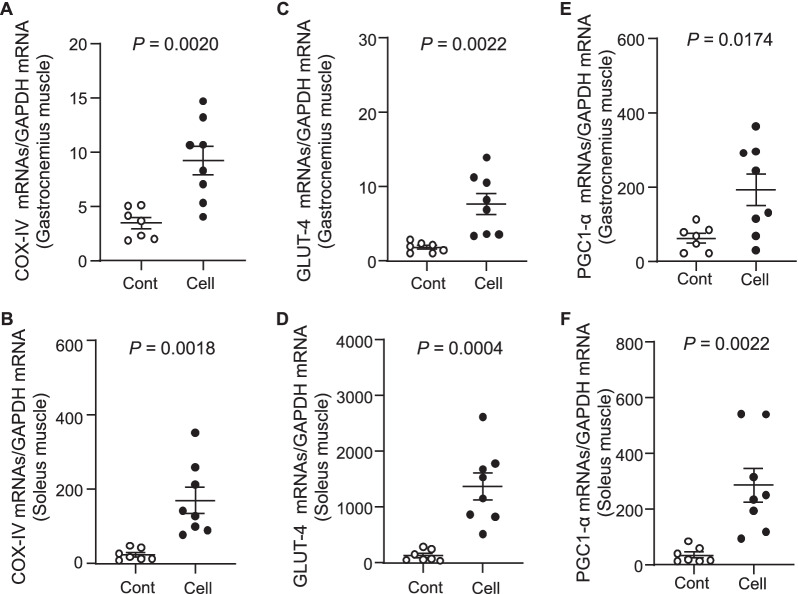
Effects of UC-MSCs on muscle mitochondrial function in 36-week-old SAMP10 mice. **A**–**F** The mRNA expression levels of cytochrome c oxidase (COX)-IV **(**A,B**)**, glucose transporter-4 (GLUT-4; C,D), and peroxisome proliferator-activated receptor-γ coactivator1-α (PGC1-α; E,F) in gastrocnemius muscle and soleus muscle were measured by quantitative RT-PCR. Data are mean ± SEM (Cont, n = 7; Cell, n = 8)

As seen in Fig. 6A, severe degeneration in the cristae of mitochondria had occurred in gastrocnemius muscles of the SAMP10 mice, as observed in by electron microscopy. The percentages of damaged mitochondria and lipid droplets were significantly lower in the UC-MSC-treated group compared to the control group (Fig. 6). The quantitative RT-PCR also revealed that the levels of COX-IV gene (Fig. 7A,B), a key enzyme of the respiratory chain, and the GLUT-4 gene expression (Fig. 7C,D) were higher in both types of skeletal muscle in the UC-MSC-treated SAMP10 mice. Moreover, PGC1-α activation has been shown to be involved in mitochondrial remodeling via the AMPK/Sirt1 axis in response to diabetes medication therapy [25]. Here, the application of UC-MSCs resulted in increased protein (Fig. 4B) and mRNAs levels of PGC1-α from both types of skeletal muscle (Fig. 7E,F).

### UC-MSCs mitigated inflammation and apoptosis in aged skeletal muscles

**Fig. 8:**
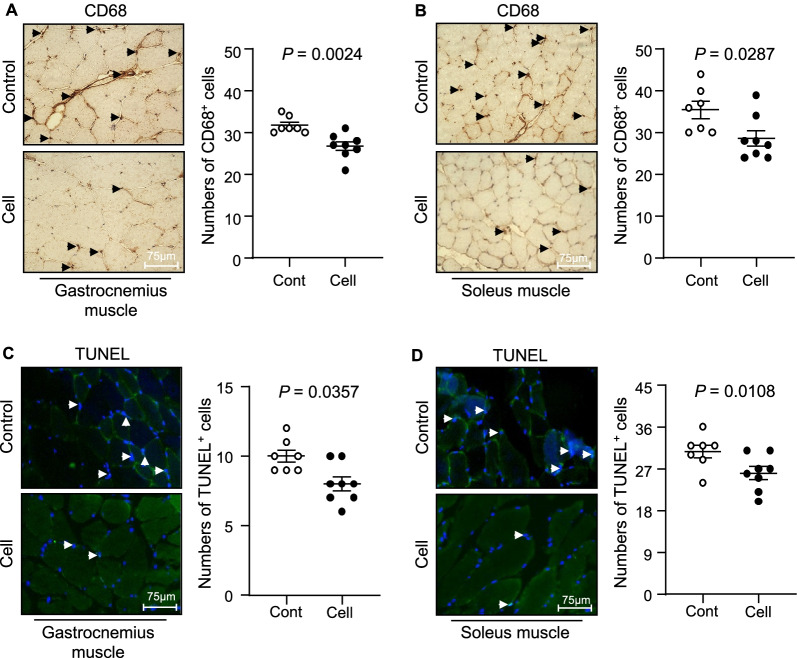
Effects of cell treatment on muscle inflammation and apoptosis in 36-week-old mice. **A**, **B** Representative CD68 immunostaining with the mouse mAb used to assess the content of macrophages and quantitative data for CD68-positive cells. **C**, **D** Representative TUNEL staining used to assess the content of apoptotic cells and quantitative data for TUNEL-positive cells. Data are mean ± SEM (Cont, n = 7; Cell, n = 8). Scale bar: 75 μm

**Fig. 9:**
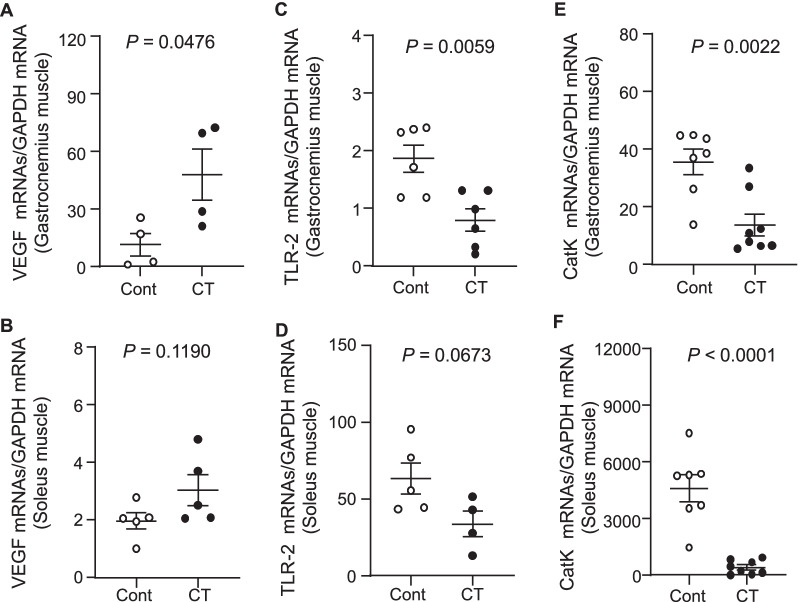
Effects of cell treatment on muscle investigated molecules in 36-week-old mice. **A**–**F**: The mRNA expression levels of vascular endothelial growth factor (VEGF; A,B), toll-like receptor-2 (TLR-2; C,D), and cathepsin K (CatK; E,F) in gastrocnemius muscle and soleus muscle were measured by quantitative RT-PCR. Data are mean ± SEM (n = 4–8)

Tissue inflammatory actions occur in a wide range of aged tissues. We thus also investigated the recruitment of macrophages, several inflammatory cytokines, and growth factors that are known to be closely associated with the aging process. Our histological observations indicated a reduced infiltration of CD68^+^ cells in the gastrocnemius and soleus muscles of 36-week-old UC-MSC-treated SAMP10 mice compared to the control group (Fig. 8A,B). Likewise, the UC-MSC injection decreased the expression of TNF-α (Fig. 5C,D) and MCP-1 (Fig. 5E,F) in both types of skeletal muscle. With the exception of the VEGF and TLR-2 levels in soleus muscle, the UC-MSC injection exerted a beneficial effect on the levels of VEGF, TLR-2, and CatK in both gastrocnemius and soleus muscle (Fig. 9).

**Fig. 10:**
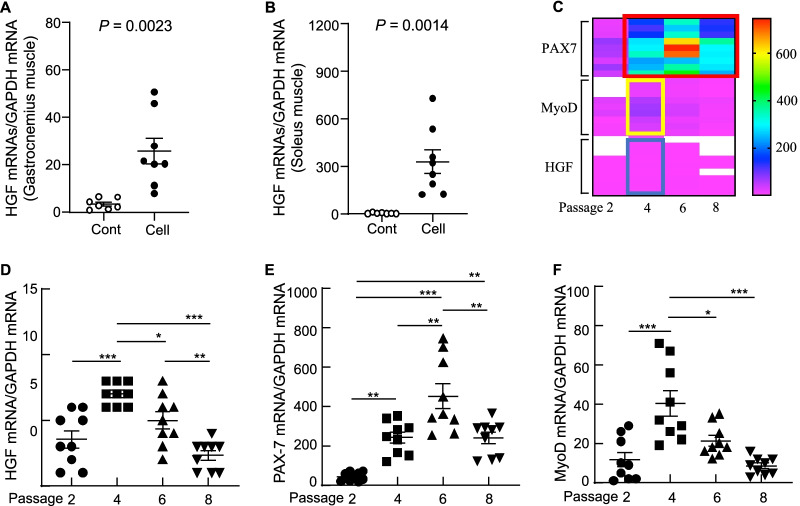
Cell treatment upregulated growth factors in both types of skeletal muscle in 36-week-old SAMP10 mice. **A**, **B** mRNA expression levels of hepatic growth factor (HGF) in gastrocnemius muscle (A) and soleus muscle (B) were measured by quantitative RT-PCR (Cont, n = 7; Cell, n = 8). **C** Heat map representing the gene expressions of paired box-7 (PAX-7), myogenic differentiation antigen (MyoD), and HGF among all cell passages (P) (P2, P4, P6, P8). Analyzed clusters, identified based on hierarchical gene clustering, are highlighted in colored boxes: *red box* for PAX-7 overexpressed in P4, P6, and P8; *yellow box* for MyoD overexpressed in P4; *blue box* for HGF overexpressed in P4. **D–F** Quantitative RT-PCR of the mRNA expression levels of HGF, PAX-7, and MyoD among all experimental cell passages (n = 9 for each group). Data are mean ± SEM

**Fig. 11:**
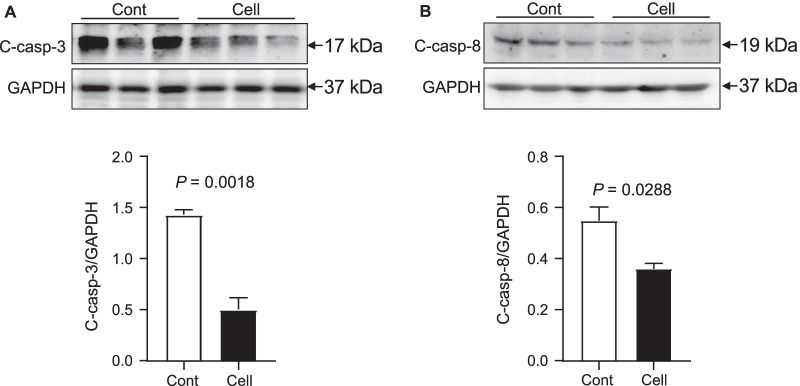
The effects of cell treatment on apoptosis-related protein expression in 36-week-old mice. **A**, **B** Representative immunoblots and quantitative analysis of Western blots for the levels of cleaved caspase-3 (C-casp-3) and cleaved caspase-8 (C-casp-8) in the gastrocnemius muscles of two groups. The expression level of each targeted protein was normalized with a Western blot antibody to GAPDH. Data are mean ± SEM (n = 3)

In agreement with the above findings of inflammation amelioration, the treatment with UC-MSCs also enhanced the expression of anti-inflammatory and antifibrotic HGF in the gastrocnemius and soleus muscles (Fig. 10A,B). As apoptosis appears to be closely associated with age-related muscle loss,[5] we tested the effects of UC-MSCs on apoptotic cells in both types of skeletal muscle. At 3 months post-cell treatment, we observed that the UC-MSCs diminished the numbers of TUNEL^+^ cells in the gastrocnemius and soleus muscles (Fig. 8C,D). As anticipated, the UC-MSC treatment lowered the levels of C-casp-3 and C-casp-8 in the gastrocnemius muscle (Fig. 11).

### UC-MSCs improved the skeletal muscle regeneration in SAMP10 mice

**Fig. 12:**
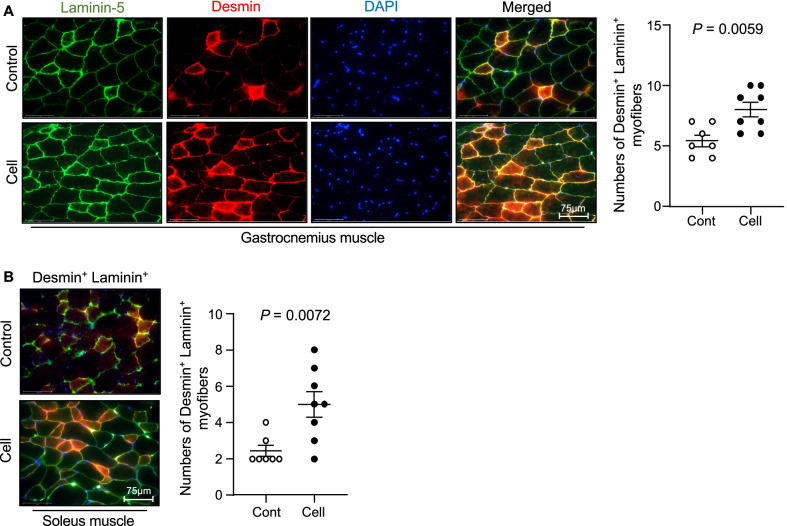
UC-MSCs ameliorated the laminin 5 and desmin expressions in the soleus and gastrocnemius muscles at 3 months post-cell treatment. **A**, **B** Representative images and quantitative data for laminin 5 (*green*) and desmin (*red*) in the gastrocnemius muscle (**A**) and soleus muscle (**B**). Data are mean ± SEM (Cont, n = 7; Cell, n = 8). Scale bar: 75 μm

**Fig. 13:**
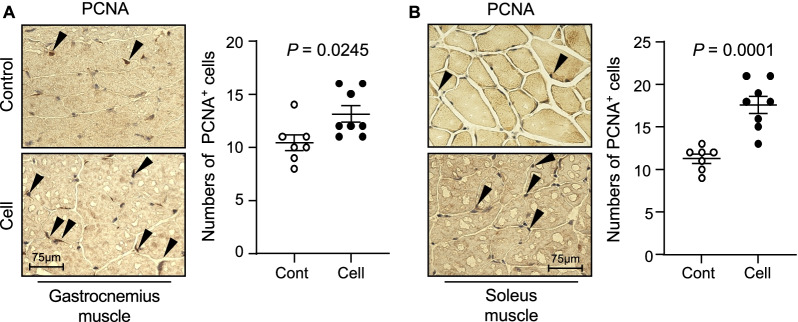
Effects of cell treatment on cell proliferation in 36-week-old mice. **A**, **B** Representative immunostaining images and quantitative data for proliferating cell nuclear antigen (PCNA)-positive cells. Data are mean ± SEM (Cont, n = 7; Cell, n = 8). Scale bar: 75 μm

**Fig. 14:**
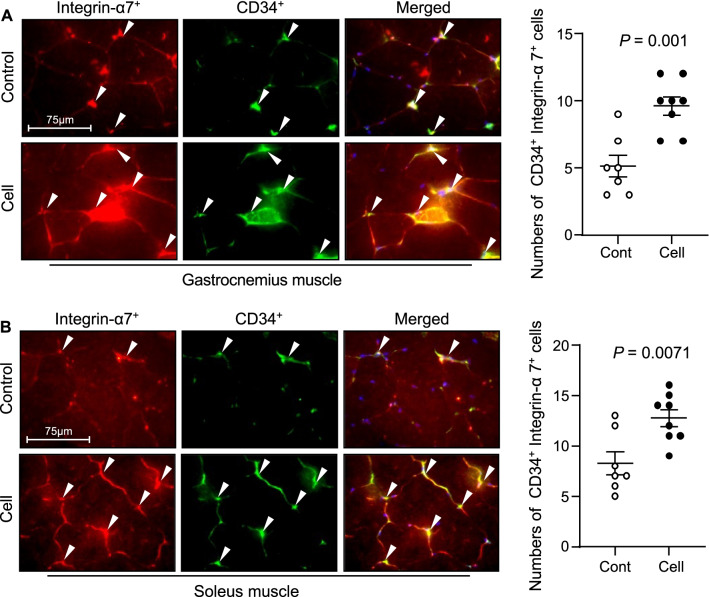
Effects of cell treatment on muscle stem cells in 36-week-old mice. **A**, **B** Representative double immunofluorescent images (**A**) and quantitative data (**B**) for CD34^+^/integrin α_7_^+^ cells. Data are mean ± SEM (Cont, n = 7; Cell, n = 8). Scale bar: 75 μm

**Fig. 15:**
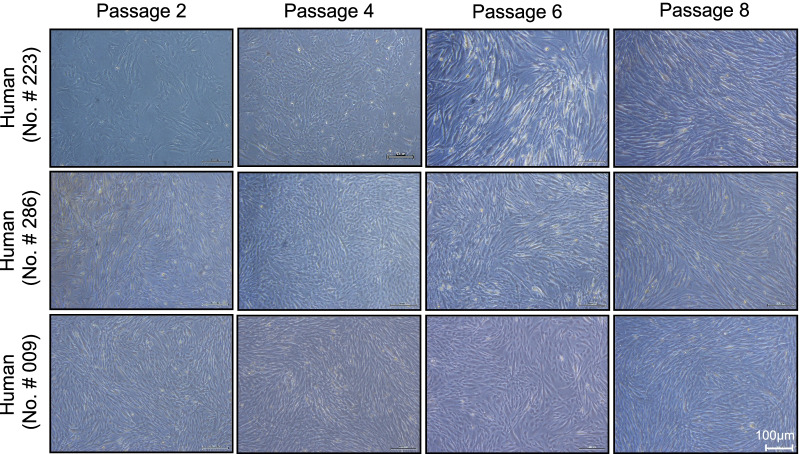
Morphology of the umbilical cord-derived mesenchymal stem cells (UC-MSCs) in the early, middle, and late phases. **A** Representative phase-contrast micrographs showed that there were many small, raised cells with a fibroblast-like appearance in the early phase, and then more and more cells appeared to be irregular and gained flat morphology in the 6th and 8th passages. Scale bar: 100 μm

To visualize the muscle repair process, we used double immunofluorescence to examine the expressions of laminin 5 (a basement membrane protein that was disorganized in immature muscle fibers of the gastrocnemius and soleus muscles of the control mice) and desmin (an intermediate filament protein that was expressed at a lower level in both types of muscle in the control mice) (Fig. 12). At 3 months post-UC-MSC treatment, both types of skeletal muscle showed strong desmin and laminin 5 expressions and ordered organization. Both UC-MSC-treated muscles had increased numbers of PCNA^+^ and CD34^+^/integrin α_7_^+^ cells (Figs. 13, 14). Similarly, the heat map and quantified data of RT-PCR showed that the expressions of biomarkers of muscle stem cells (i.e., PAX-7, MyoD, and HGF) were much higher at the 4th passage of the UC-MSCs (Fig. 10C–F). Moreover, the representative phase-contrast micrographs revealed that although there were only many small, raised cells with a fibroblast-like appearance in the early phase, more and more irregular and flat cells appeared in the later (6th and 8th) passages of the UC-MSCs (Fig. 15). In addition, the immunostaining with anti-human dystrophin antibody showed that there was no positive staining signal in the gastrocnemius or soleus muscles (data not shown).

## Discussion

**Fig. 16:**
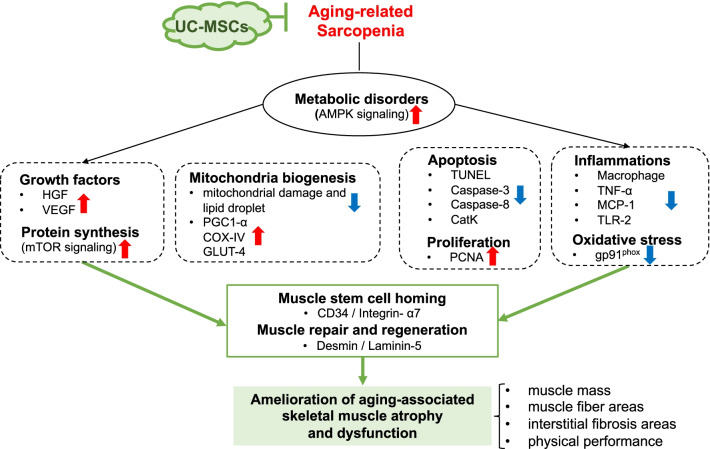
Proposed mechanism of UC-MSC-mediated alleviation of muscle mass loss and dysfunction in a SAMP10 mouse model. UC-MSCs: umbilical cord-derived mesenchymal stromal cells, AMPK: adenosine monophosphate (AMP)-activated protein kinase, mTOR: mammalian target rapamycin, HGF: hepatocyte growth factor, VEGF: vascular endothelial cell growth factor, PGC-1α: peroxisome proliferator-activator-γ coactivator-1α, MCP-1: monocyte chemoattractant protein-1, COX-IV, cytochrome c oxidase subunit 4; GLUT-4, glucose transporter-4; TUNEL, terminal deoxynucleotidyl transferase-mediated dUTP nick end labeling; CatK, cathepsin K; PCNA, proliferating cell nuclear antigen; TNF-α: tumor necrosis factor-alpha, TLR-2, toll-like receptor-2

Over the past decade, the therapeutic effects of UC-MSCs in various pathophysiological conditions have been revealed by clinical and experimental studies [12, 13]. Those and other studies evaluated the safety and potential efficacy of an allogeneic human UC-MSC infusion in acute brain and lung injuries [12, 21]. Herein, we examined the therapeutic effect and potential mechanism of the use of UC-MSCs in the aging-associated skeletal muscle atrophy of SAMP10 mice. The five principal findings of our study are as follows. (1) The mice that underwent cell treatment as early as at 24 weeks of age showed ameliorated muscle mass loss and improved physical performance compared to the control non-treated mice. (2) UC-MSCs preserved not only the AMPK signaling activation but also the mTOR-mediated protein synthesis in skeletal muscles of SAMP10 mice. (3) Simultaneously, the UC-MSCs ameliorated the mitochondrial dysfunction and activation of PGC1-α, which is a key protein for mitochondrial biogenesis in skeletal muscle. (4) The UC-MSC treatment retarded the oxidative stress production of a key enzyme, i.e., the nicotinamide adenine dinucleotide-phosphate oxidase subunit (gp91^phox^) expression, and it mitigated apoptosis and inflammatory actions in skeletal muscles. (5) The therapeutic intervention using UC-MSCs stimulated the repair of skeletal muscle, accompanied by increased numbers of PCNA^+^ proliferating cells and CD34^+^/integrin α_7_^+^ muscle stem cells in SAMP10 mice. The potential mechanisms underlying the improvement of aging-related loss of muscle mass and function in a SAMP10 mouse model are schematically represented in Fig. 16.

Accumulating evidence suggests that several types of treatment with MSCs were beneficial against various diseases, e.g., ischemic cardiovascular–cerebral disorders and muscle atrophies [15, 26, 27]. The data obtained in the present study demonstrate that the UC-MSC treatment improved the grip strength and endurance of SAMP10 mice. The UC-MSCs also preserved the muscle fiber areas and reduced the interstitial fibrosis areas in the gastrocnemius and soleus muscles of SAMP10 mice. We observed that UC-MSCs markedly enhanced the levels of protein synthesis-related molecules (p-mTOR, p-Erk1/2, and sMHC), suggesting that the use of UC-MSCs is likely to improve muscle integrity via the activation of an mTOR-Erk1/2 signaling pathway in the aged skeletal muscles of SAPM10 mice.

Skeletal muscle is a mitochondrion-rich tissue, accounting for approx. 80% of the postprandial blood glucose uptake in the normal state [28]. Enhancing the mitochondrial biogenesis of skeletal muscle facilitates the improvement of muscular strength [7, 29]. Mitochondrial biogenesis is considerably well regulated by PGC1-α, which promotes mitochondrial nuclear gene transcription and increases the number of mitochondria [30]. In accord with the accumulating evidence indicating that the expression of PGC1-α in skeletal muscle declines with age and various metabolic diseases [31], we speculate that the effects of UC-MSCs on PGC-1α activity are likely to be of great significance in our experimental setting. Our present findings revealed that UC-MSCs not only retarded age-associated mitochondrial damage and lipid droplet accumulation but also induced the expressions of PGC1-α and COX-IV. Thus, the ability of UC-MSCs to improve mitochondrial biogenesis has a salutary effect on the skeletal muscles' intrinsic function, even under conditions of aging-related oxidative stress. Other studies have shown that (*i*) AMPK can upregulate the activity of PGC1-α by increasing the intracellular concentration of NAD^+^ to activate silent information regulator 1 (Sirt1), and (*ii*) this can ultimately promote mitochondrial biogenesis [32, 33]. It was reported that AMPK/Sirt1/PGC-1α activation accelerated the mitochondrial morphological changes and its dysfunctions in rat muscles [34]. Taking these findings together with our present observations of UC-MSC-induced AMPKα phosphorylation, we propose that the PGC1-α overexpression-mediated mitochondrial biogenic capacity and the rescue of mitochondrial injury are likely to be attributable, at least in part, to the enhancement of the AMPK activation.

In agreement with a report that MSCs reduced endothelial cell apoptosis in ischemic muscles at advanced ages [35], our present TUNEL staining results demonstrated that cell apoptosis in the gastrocnemius and soleus muscle were reduced by UC-MSC treatment. We observed decreased levels of gp91^phox^ gene and apoptosis-related proteins (C-casp-3, C-casp-8, and CatK) in both types of skeletal muscle in SAMP10 mice. As AMPK has been reported to protect against cardiac cell and muscle apoptosis [34, 36], UC-MSCs appear to prevent muscle loss via AMPK-mediated anti-apoptotic and anti-oxidative actions in SAMP10 mice. Moreover, aging and metabolic disorders cause increases in the levels of inflammatory cytokines in tissues [37], and we observed herein that treatment with UC-MSCs resulted in decreased inflammatory actions in SAMP10 mice. Our results revealed that the UC-MSC treatment resulted in decreased expression levels of TNF-α, MCP-1, TLR-2, and CatK genes in mouse gastrocnemius and soleus muscles, and this was further supported by the immunostaining results, i.e., the UC-MSC intervention diminished the infiltration of CD68^+^ macrophages in both types of skeletal muscle in SAMP10 mice. Taken together, our findings indicate that UC-MSCs protect muscle cells against apoptosis and inflammation and may therefore modulate the ability of skeletal muscle cells to induce muscle repair and/or regeneration, thereby preventing aging-related muscle dysfunctions.

It has been clear that VEGF and HGF modulate bone marrow stem cells the mobilization and modification of them to contribute cardiovascular and muscle tissue regeneration [5, 38–42]. A recent review highlighted that cell regenerative events in geriatric tissue healing are controlled by the growth factors including HGF that are activated by an injury [43]. It was reported that skeletal muscle tissues also have stem cells (called satellite cells), but they are present at low levels, and new muscle fibers that are derived from them may not be enough to replace injured fibers [44]. Bone marrow MSCs could promote musculoskeletal tissue regeneration and activate the myogenic differentiation of satellite cells [39]. Rejuvenation of the CD45^−^CD31^−^ CD11b^+^Sca-1^−^CD34^+^integrin α_7_^+^ muscle stem cell (MuSC) population restores strength to injured aged muscles [45]. We have demonstrated that aging impaired bone marrow-derived CD34^+^integrin α_7_^+^ MuSC mobilization and homing to the skeletal musculature in SAMP10 mice, and these changes were rectified by long-term exercise [5]. Apoptotic cell-derived proliferin-1 has been shown to trigger bone marrow CD34^+^integrin α_7_^+^ MuSC mobilization to contribute to muscle repair in mice in response to a cardiotoxin [46]. In the present study, the expression of HGF and VEGF genes was higher in the cell-treated mice compared to the control mice, and double immunofluorescence demonstrated that UC-MSC treatment enhanced the numbers of CD34^+^integrin α_7_^+^ cells in the gastrocnemius and soleus muscles. We also observed that the skeletal muscles of the UC-MSC-treated mice showed strong desmin and laminin 5 expressions and ordered organization. Collectively, the above-described findings indicate that the ability of UC-MSCs to increase HGF and VEGF levels in SAMP10 mice is likely to contribute to a triggering of bone marrow-derived MuSC mobilization and homing into the injured musculature, leading to the improvement of aging-related muscle regeneration and dysfunction. Earlier studies demonstrated that in lung and brain injury models, intravenously injected UC-MSCs were engrafted mainly in lung tissues until 2 days post-injection and then disappeared by day 4; no cell-labeled signal was observed in targeted or non-targeted whole body tissues at day 7 [12, 21]. In our present investigation, we did not detect human dystrophin-positive staining in the gastrocnemius or soleus muscles of UC-MSC-treated SAMP10 mice (data not shown). These findings suggest the possibility that the UC-MSC-mediated skeletal muscle benefit might be attributable to the paracrine effect rather than injected cell homing and differentiation to skeletal muscles.

There are several study limitations. First, although accumulating evidence indicates that after an injection of UC-MSCs in wild-type mice it is difficult for the UC-MSCs to engraft and locate in skeletal muscle tissues, we were not able to use representative bioluminescence overlay images or a green fluorescent protein (GFP)-labeled method to determine whether the UC-MSCs were engrafted into the muscle after their injection in our model mice. Second, we did not obtain direct evidence regarding whether and where the injected UC-MSCs were differentiated into the skeletal muscle cells in SAMP10 mice. In vitro, the special commercial differentiation medium used in all of the present experiments did not trigger UC-MSC differentiation into myotubes. Third, we could not fully explore the effect of UC-MSCs on all types of fibers in gastrocnemius and soleus muscles. Lastly, unfortunately, this study was not designed to explore the magnitude of the functional, morphological, or biochemical deficiencies between SAMP10 and normal healthy mice, or to determine to what extent the cell treatments contribute to normalizing these characteristics. Further research is necessary to investigate these issues. We hope that the information gained herein will contribute to the development of additional in vivo models for evaluating the use of UC-MSCs in various clinical applications.

## Conclusions

UC-MSCs stimulated the response of mouse muscles in pathological conditions, including sarcopenia. We performed translational studies using UC-MSCs toward the goal of developing therapies for aging-related muscular disease. The results of this study indicate that aging-associated muscular loss can be ameliorated by treatment with UC-MSCs, which might be due to the improvement of oxidative stress production, apoptosis, and inflammation as well as AMPK/PGC1-α-signaling-mediated mitochondrial biogenesis. The ability of UC-MSCs to restore the young-muscle response should be further investigated as a powerful strategy to counteract aging-associated declines in muscle repair and/or regeneration by paracrine factors. Such investigations might also enable the therapeutic use of stem cell technology to replace or complement pharmacological interventions or physical exercise for aging individuals with sarcopenia and frailty.

## Data Availability

All data generated or analyzed during this study are included within the article. (2) All data of this study are available from the corresponding author upon request.

## Abbreviations

COX-IV: Cytochrome c oxidase subunit 4
GAPDH: Glyceraldehyde 3-phasphate dehydrogenase
GLUT-4: Glucose transporter-4
HGF: Hepatocyte growth factor
MCP-1: Monocyte chemoattractant protein-1
MyoD: Myogenic differentiation antigen
PAX-7: Paired box-7
PGC1-α: Peroxisome proliferator-activated receptor-γ coactivator1-α
TNF-α: Tumor necrosis factor-α
TLR-2: Toll-like receptor-2
CatK: Cathepsin K
VEGF: Vascular endothelial growth factor
